# Small and Micro-Water Quality Monitoring Based on the Integration of a Full-Space Real 3D Model and IoT

**DOI:** 10.3390/s24031033

**Published:** 2024-02-05

**Authors:** Yuanrong He, Yujie Yang, Tingting He, Yangfeng Lai, Yudong He, Bingning Chen

**Affiliations:** 1 Big Data Institute of Digital Natural Disaster Monitoring in Fujian, Xiamen University of Technology, Xiamen 361024, China; heyuanrong@126.com (Y.H.); 2222041004@s.xmut.edu.cn (T.H.); l15280367516@163.com (Y.L.); haoduosci@163.com (Y.H.); 13394716601@163.com (B.C.); 2 Hunan Key Laboratory of Remote Sensing Monitoring of Ecological Environment in Dongting Lake Area, Changsha 410004, China

**Keywords:** water quality monitoring system, IoT, full-space real 3D model, multi-source data

## Abstract

In order to address the challenges of small and micro-water pollution in parks and the low level of 3D visualization of water quality monitoring systems, this research paper proposes a novel wireless remote water quality monitoring system that combines the Internet of Things (IoT) and a 3D model of reality. To begin with, the construction of a comprehensive 3D model relies on various technologies, including unmanned aerial vehicle (UAV) tilt photography, 3D laser scanning, unmanned ship measurement, and close-range photogrammetry. These techniques are utilized to capture the park’s geographical terrain, natural resources, and ecological environment, which are then integrated into the three-dimensional model. Secondly, GNSS positioning, multi-source water quality sensors, NB-IoT wireless communication, and video surveillance are combined with IoT technologies to enable wireless remote real-time monitoring of small and micro-water bodies. Finally, a high-precision underwater, indoor, and outdoor full-space real-scene three-dimensional visual water quality monitoring system integrated with IoT is constructed. The integrated system significantly reduces water pollution in small and micro-water bodies and optimizes the water quality monitoring system.

## 1. Introduction

In recent years, the development of the park has increasingly focused on ecological environment construction, and the construction of small and micro-water bodies has become a necessary option. Small and micro-water bodies are mostly enclosed landscape water bodies characterized by a small catchment area, no water circulation, weak self-purification capacity, and high susceptibility to external pollution [1]. In addition, they are located in densely populated areas, which means that the decline in water quality not only affects the overall ecological environment of the park but also poses hidden risks to the health of park personnel [2]. Therefore, in order to reduce water pollution, a large number of scholars have carried out research on water quality monitoring systems. Huibo Zhu et al. designed a remote water parameter monitoring and management system using WIFI as a wireless communication mode and the Alibaba Cloud platform as a network medium [3]. Zhihao Fang designed a lightweight water quality monitoring node based on NB-IOT and built a water quality supervision platform by using the Spring + Spring MVC+MyBatis framework [4]. Yongxiong Zeng used NB-IoT to transmit water quality data to the cloud transparent transmission platform, evaluated water quality based on a random forest algorithm, and realized functions such as collection and processing, wireless transmission, storage analysis, and visual display of industrial water pollution data [5]. The above scholars built a wireless remote real-time water quality supervision system through various technologies, realized wireless remote real-time monitoring of water quality information, and effectively reduced water pollution. However, in the small and micro-water ecological restoration, many scholars also put forward more requirements. Du Bing et al. first explored the key points of small and micro-wetlands and their water ecological restoration and then emphasized that water ecological restoration should follow the integrity principle by combining various elements such as wetland topography, water system structure, and wetland organisms [6]. Xu Xin et al. put forward the correlation analysis of variables based on DEM elevation data, the general situation of small and micro-water bodies, precipitation distribution data, the number of permanent urban residents in each district of Huai ‘an, and the number of permanent residents at the end of the year by using SPSS19 software and made it clear that water quality parameters were the basis for evaluating whether water quality restoration was up to standard [7]. It can be seen that natural resources data, geographical and topographic data, hydrometeorological data, and water quality parameters are the basis for planning the construction, restoration, and management of small and micro-water bodies. The simple water quality supervision system based on the Internet of Things can no longer meet the needs of water quality supervision and ecological restoration.

Since 2020, the national authority in charge of natural resources, surveying, mapping, and geographic information has been vigorously promoting the construction of a 3D China with real-world scenes, encouraging the expansion of more application areas for full-space real-world scenes. The full-space real-world model carries rich terrain geographic information and natural resources and can realize the real, 3D, and time-sequential mapping of human production, life, and ecological space, with the potential to overcome the problems of information deficiency, one-sidedness, and abstraction in traditional two-dimensional geographic systems [8,9]. Meanwhile, the Internet of Things has been able to achieve wireless remote real-time monitoring, positioning tracking, and other functions through multiple sources of sensors, video surveillance, the Global Navigation Satellite System (GNSS), and wireless communication technology. It has successfully integrated and expressed dispersed, real, and warning-enabled environmental IoT sensing data [10,11]. In order to address the problem of deteriorating water quality in small and micro-water bodies due to poor circulation and complex environments, as well as the need to restore the ecological balance of these water bodies, a large amount of geographic and terrain information, natural resource information, and meteorological and water resources information must be taken into account.

This study first relies on various new geographic information products to construct a full-space real-world 3D model base of “above and below ground, indoor and outdoor, and water surface and bottom”. Secondly, the Internet of Things technologies, such as GNSS, multi-source sensors, video surveillance, and NB-IoT wireless communication, are utilized to design low-power, green, and environmentally friendly water quality monitoring hardware terminals. Finally, a 3D visualization water quality monitoring system is constructed based on the Cesium framework. The system provides users and managers with a more real and three-dimensional water quality monitoring experience, effectively realizes the spatial data of water quality, integrates spatial information data, and improves the small and micro-water pollution problems.

## 2. Water Quality System Architecture

**Figure 1:**
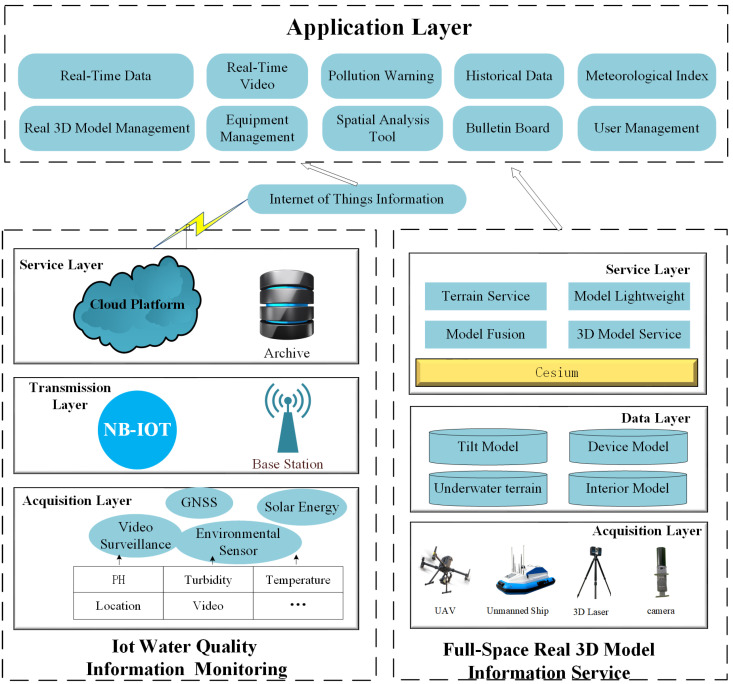
Water quality system architecture.

Based on the system requirements, the design and implementation are carried out from the real-time monitoring of water quality information of the Internet of Things, 3D information service of full space reality, and system application layer. The system architecture is shown in Figure 1.

(1) IoT Water Quality Information Real-Time Monitoring

IoT water quality information real-time monitoring is mainly responsible for the acquisition, transmission, and management of water quality data. In the acquisition layer, the STMF32 microcontroller serves as the low-power core of the whole water quality data acquisition layer, which is used to process the sensing data from PH value, turbidity, and temperature sensors and receive GNSS positioning information. In addition, the controller uses video monitoring with remote lighting, the water environment, and water quality monitoring equipment for 24 h a day video monitoring. The transmission layer uses NB-IoT for wireless remote transmission to realize real-time monitoring of water quality information by the client. The OneNet cloud platform is selected for the service layer, which is responsible for data acquisition, classification, and storage.

(2) Full-Space Real 3D Model Information Service

Full-space 3D information service mainly includes the acquisition layer, data layer, and service layer. A multi-technology complementary approach is used to collect the full-space data, in which the UAV tilt photography technology can obtain outdoor terrain data and building image data in a wide range. Three-dimensional laser scanning technology was used to obtain indoor point cloud data. The underwater terrain data are obtained by the unmanned ship measurement system. Combined with the near-ground image measurement technology, the details are supplemented to improve the quality of the 3D model. Professional tools such as ConTextCapture, DP-modeler, and SketchUp were used for data preprocessing and fine modeling of the acquired image data. Finally, the constructed inclined model, indoor model, and underwater terrain model were lightweight, fused, and service released through the Cesium platform.

(3) Application Layer

The application layer is primarily responsible for integrating and displaying various data sources and carrying out various applications. Specifically, the application layer can provide 3D visual geographic information scenes and has basic base map operations such as real 3D scene browsing, measurement, query, and map conversion. On this basis, the water quality information is displayed dynamically in the form of charts and dashboards in real time, functions such as early warning analysis and historical data management are added, and real-time video surveillance is connected to monitor the state of the water environment in an all-around way.

## 3. Iot Water Quality Monitoring Design

### 3.1. Hardware Terminal Circuit Design

**Figure 2:**
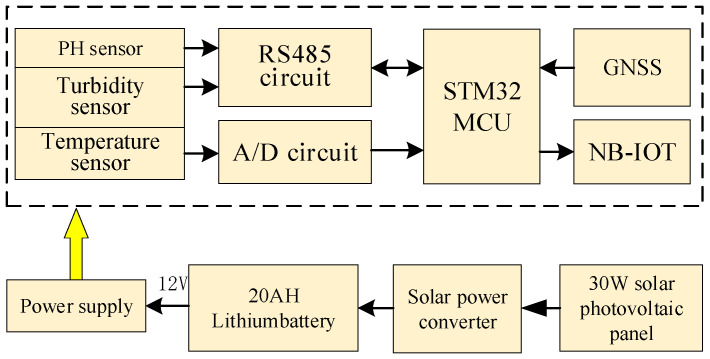
Water quality monitoring hardware terminal design block diagram.

In this paper, low-power STM32F103ZET6(STMicroelectronics, Geneva, Switzerland) is used as the main controller, integrated power circuit, water quality acquisition circuit, communication circuit, and GNSS positioning circuit for collecting water quality information. Since the water quality monitoring device has been floating on the water body for a long time, the energy supply of the water quality monitoring device is limited, so the combination of solar photovoltaic panels and lithium batteries is used to power the monitoring device to achieve self-sufficiency in electric energy. The water quality monitoring hardware terminal design block diagram is shown in Figure 2.

#### 3.1.1. Power Circuit Design

**Figure 3:**
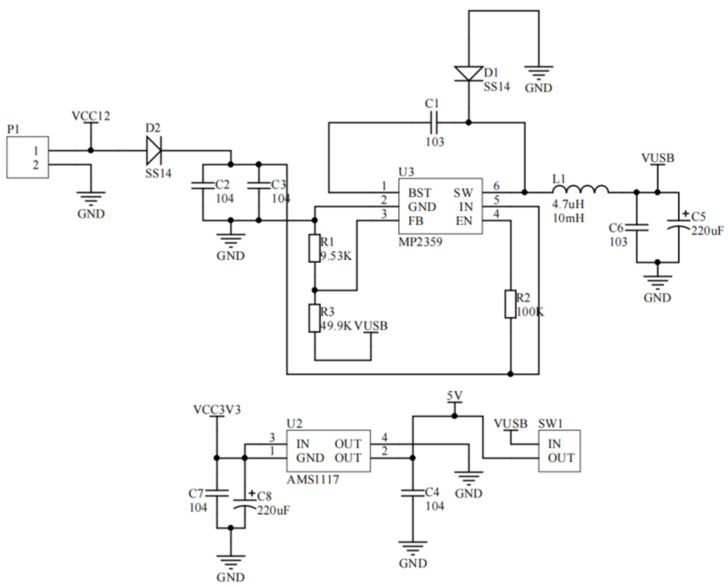
Power circuit diagram.

The startup voltage varies among different modules, but the output voltage of a conventional lithium battery is 12 V. Therefore, to meet the power supply requirements of different sensor modules, this paper needs to redesign the power supply circuit. Specifically, the 12 V voltage is first converted to 5 V through the chip MP2359, where the capacitor C1 is connected to the floating power supply at the BST and SW ends to drive the chip switch, and a 100 kΩ resistor is connected between the EN pin and the IN pin to pull the EN above 1.2 V to enable the chip automatically. Two parallel capacitors with 0.1 μF capacitors are connected to the IN end to achieve the function of input and output filtering and avoid the instability of voltage signals that may cause chip burnout. Then, the AMS1117 chip is used to convert the 5 V voltage to 3.3 V, and the LM1117 adopts a standard four-pin linear regulator to filter the input and output through capacitors C7 and C8, avoiding the circuit problems caused by the instability of the output voltage. The power supply circuit is shown in Figure 3.

#### 3.1.2. Water Quality Acquisition Circuit Design

The design of the water quality acquisition circuit is to complete the data acquisition work of each sensor. The RS-PH-N01-3 PH sensor (Shandong Renke Control Technology Co., Ltd., Jinan, China) and RS-ZD-N01 turbidity sensor (Shandong Renke Control Technology Co., Ltd., Jinan, China) are RS485 water quality sensors that support the MODBUS-RTU communication protocol. It is suitable for a variety of water quality monitoring scenarios and has the characteristics of high precision and high stability [12]. The DS18B20(Qinhe Intelligent Technology Co., LTD., Shenzhen, China) temperature sensor is a digital water temperature sensor with a special single-wire interface so that only one interface pin is needed for communication. The main parameters of the water quality sensor are shown in Table 1.

**Figure 4:**
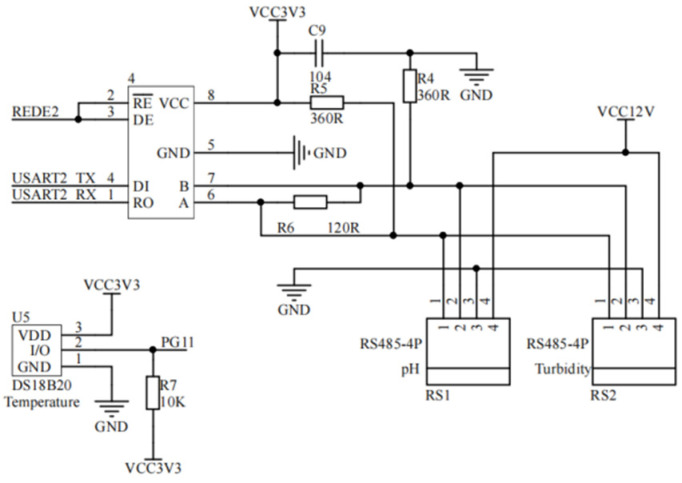
Water quality acquisition circuit diagram.

The water quality acquisition circuit uses an SP3485 chip to convert the 485 signal of the PH value and turbidity sensor to the TTL signal of the main controller. In order to ensure the stability of data transmission, the resistance R6 with an A resistance value of 120 Ω is connected in parallel between the A and B pins of the chip. To reduce the interference caused by the external current on the chip when the input end is hung, connect 360 Ω resistances R4 and R5 to ports A and B as bias resistances of the acquisition circuit. R4 is connected to the GND end as a pull-down resistor, and R5 is connected to the VCC end as a pull-up resistor. The RE and DE pins are connected to REDE2 of the main controller. When the single-chip microcomputer wants to send data, the control DE is high, RE is low, and the data are sent out through TXD. When the microcontroller wants to receive data, the control DE is low, RE is high, and the data are received back through RXD to complete the collection of PH value and turbidity. The I/O pin of the temperature sensor is directly connected to the PG11 pin of the main controller for temperature data acquisition. The water quality acquisition circuit is shown in Figure 4.

#### 3.1.3. NB-IOT Module Communication Circuit

**Figure 5:**
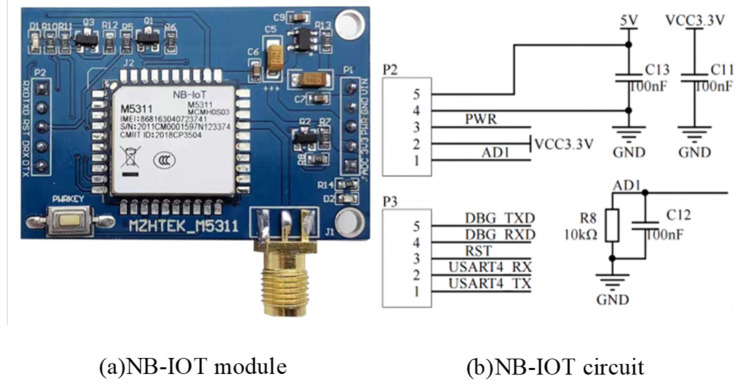
NB-IOT module circuit diagram.

The wireless communication module is the M5311 module, which is a high-performance, low-power NB-IoT wireless module based on the MT2625 platform. The module supports a variety of network protocol stacks and has the advantage of embedded SDK of mainstream Internet of Things platforms such as OneNet, Alibaba Cloud, and Tencent Cloud. It provides convenience for multi-platform connection [13,14]. NB-IoT connects the UART serial port with PB10 and PB11 of the STM32 microcontroller through NB_RX and NB_TX pins, respectively, to realize real-time transmission of water quality data. The circuit of the NB-IOT module is shown in Figure 5.

#### 3.1.4. GNSS Positioning Circuit

**Figure 6:**
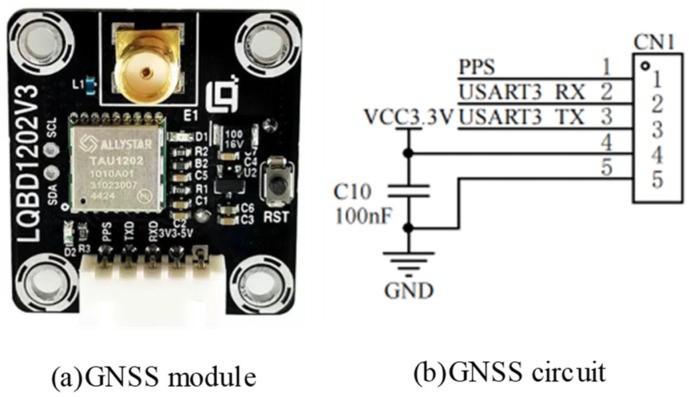
GNSS positioning module circuit diagram.

The LQBD1202 Beidou dual-frequency GNSS positioning module can receive Beidou +GPS signals at the same time to achieve sub-meter accurate positioning and supports the UART serial port communication interface, so it can directly communicate with the STM32 main controller. The circuit diagram of the GNSS positioning module is shown in Figure 6.

### 3.2. Water Quality Monitoring Software Design

**Figure 7:**
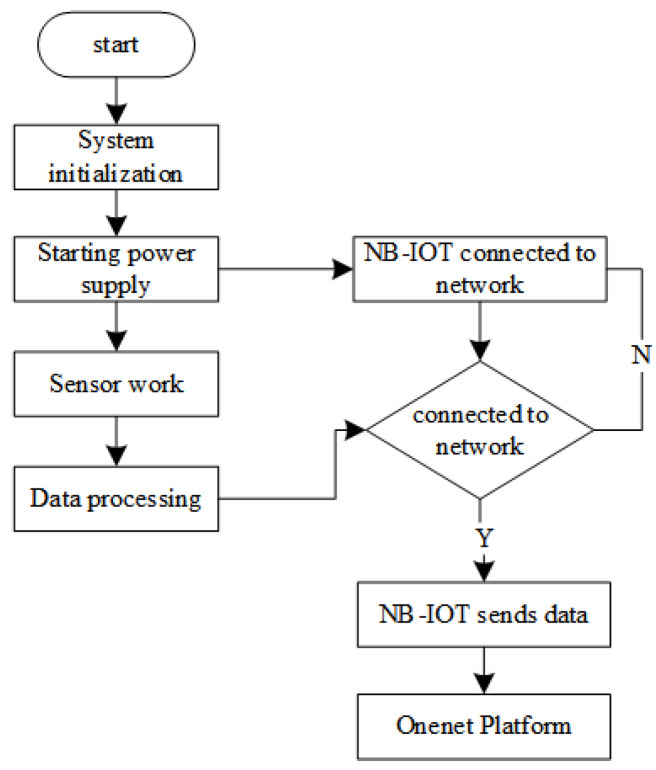
Main program flow diagram.

The software design of water quality monitoring is to realize the control management of monitoring terminal, monitoring data acquisition, and data transmission. After the hardware terminal is powered on, each module and communication interface is first initialized, and AT instructions are sent to complete the NB-IOT networking, water quality data, and GNSS positioning data to the cloud platform. After the data transmission is complete, turn off the power of the functional modules except for the main controller module, and the main controller enters the standby mode. When the time reaches the scheduled time, it automatically wakes up and enters the data acquisition state again. The software program design flow of the water quality monitoring is shown in Figure 7.

**Figure 8:**
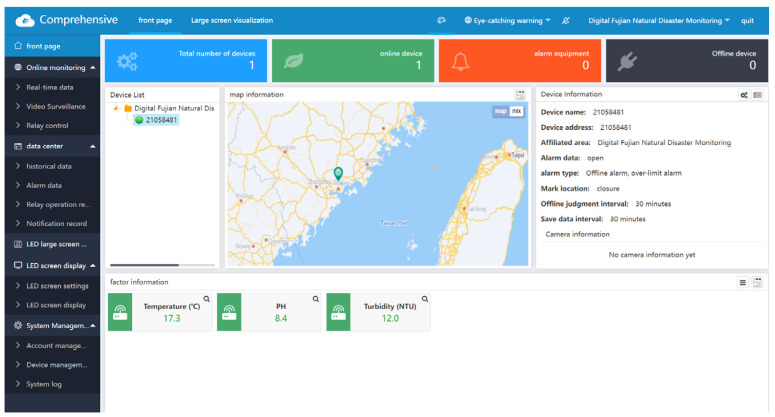
Cloud platform page diagram.

The water quality data and GNSS positioning data were received and stored on the OneNet cloud platform. The third-party client accessed the cloud platform database, downloaded the relevant data, and played back the historical data through the API key provided by OneNet. It is the IoT data management hub of the water quality monitoring system based on the integration of full-space real 3D and IoT. The cloud platform page is shown in Figure 8.

## 4. Construction of Full-Space Real 3D Based on Multi-Source Data

**Figure 9:**
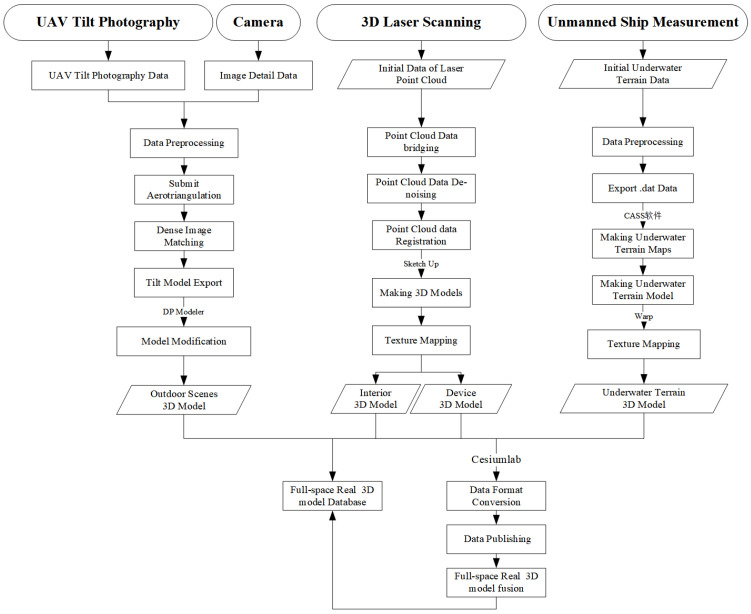
The technical flow chart of the full-space real 3D model construction.

Full-space reality 3D is a 3D model of the real world built on the basis of the real world through the acquisition, processing, and integration of multiple data sources, aiming to achieve an all-around, full-space, high-precision, and interactive display of the real world [15]. Traditional techniques for constructing 3D models include UAV tilt photography, 3D laser scanning, and close-range photogrammetry. However, due to the high density and high height of buildings, diverse architectural styles, and the occlusion of buildings’ ground levels by plants and trees within the park area, traditional 3D models often suffer from low quality and unclear textures. It is challenging for a single method to address these issues. Therefore, this study adopts a complementary approach to construct a full-space real 3D model by leveraging multiple space information acquisition techniques [16]. The technical process of full-space real 3D construction is shown in Figure 9.

### 4.1. Outdoor Real 3D Model Construction

After the outdoor tilt photography and detail photography are quickly obtained by UAV and camera, ContextCapture4.4.9.516 software and DP Modeler2.0 software are used to complete dense image matching, submit aerotriangulation, and model construction.

(1) Dense Image Matching

When a UAV takes aerial photography, the angle and position of the camera will change during flight, resulting in possible differences in position, rotation, and scale of the images captured. Therefore, it is necessary to carry out dense image matching to accurately map the image obtained by UAV tilt photography to the geographical coordinate system.

(2) Submit Aerotriangulation

Aerotriangulation can be used to calculate the three-dimensional coordinates of the target object by using the photographic parameters obtained from aerial photography, the corresponding relationship of feature points, and the principle of photographic geometry. This is to prepare for later model reconstruction.

(3) Model Construction

**Figure 10:**
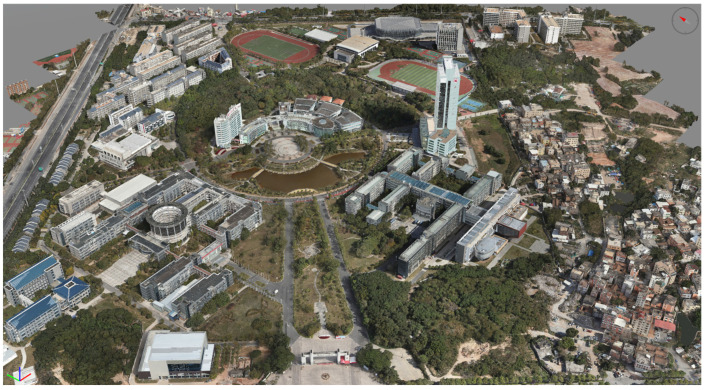
Outdoor real 3D model.

After data processing by ContextCapture software, the tilt model is automatically output, and the tilt model is imported into DP Modeler software to modify or rebuild the problem model and texture. Finally, the outdoor real 3D model in OSGB format is output. The outdoor real 3D model is shown in the following Figure 10.

### 4.2. Interior Real 3D Model Construction

Three-dimensional laser scanning technology was used to obtain the interior laser point cloud data of the natatorium. TRW12.3 software and SketchUp 2022 software are used for point cloud data de-noising, point cloud data registration, and model construction.

(1) Point Cloud DataDe-noising

Point cloud de-noising is to remove the noise points in the initial data of the laser point cloud and retain the characteristics of the point cloud as much as possible. The reflection intensity of large noise points and isolated points in point cloud data will be significantly different from that of surrounding point clouds. For such useless points, they can be removed manually or filtered by reflection intensity according to the situation.

(2) Point Cloud Data Registration

Due to the limited collection range of the 3D laser scanner, a scan can not fully cover the entire natatorium; a total of multiple sites was set up to obtain point cloud data, so it is necessary to integrate and splice the point cloud data collected by all stations. When splicing, coordinate system alignment, removal of overlapping areas, and adjustment of the matching degree between data are required to ensure the integrity and accuracy of the point cloud data after splicing. Finally, the point cloud data in the LAS format are exported.

(3) Model Construction

**Figure 11:**
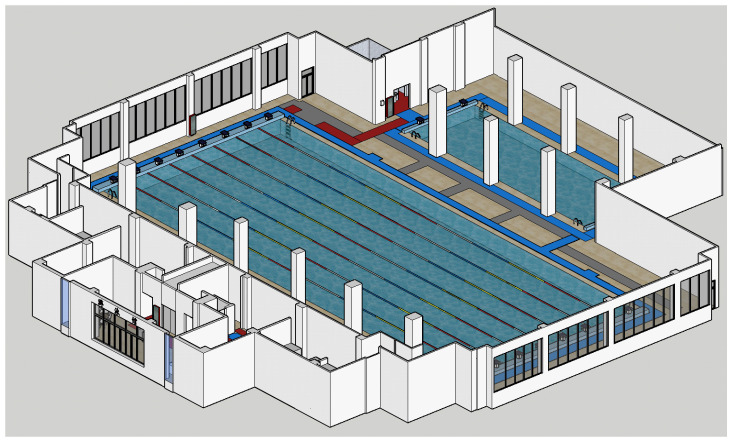
Interior real 3D model.

The processed point cloud data were imported into SketchUp software to conduct fine modeling of points, lines, and surfaces and texture mapping for the model. Finally, the interior real 3D model of the natatorium in obj format will be output. The interior real 3D model is shown in Figure 11.

**Figure 12:**
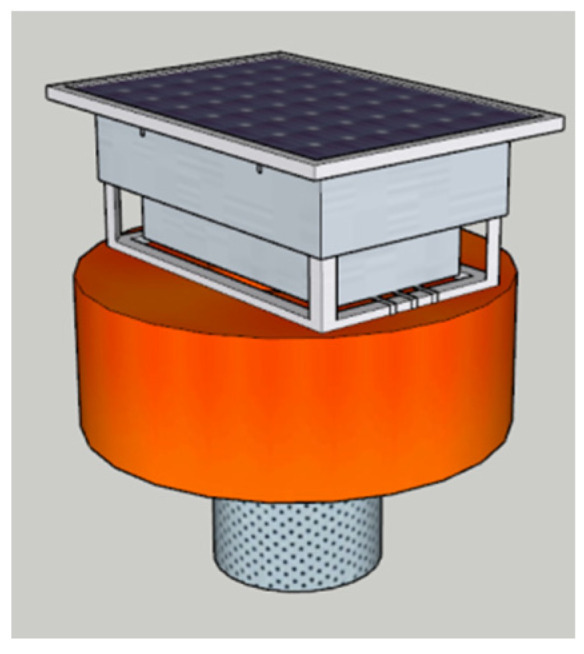
Water quality monitoring device 3D model.

For the water quality monitoring device model, SketchUp2022 software is still used to reconstruct the 3D white model at a 1:1 scale, and true color photos are taken by the camera for texture mapping. The water quality monitoring device 3D model is shown in Figure 12.

### 4.3. Underwater Terrain Real 3D Model Construction

**Figure 13:**
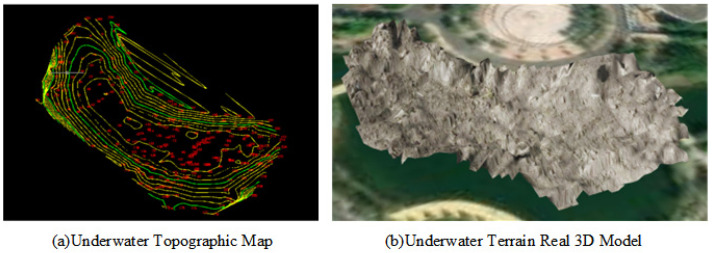
Underwater Terrain Real 3D model.

The unmanned boat measurement system is equipped with the DeepVision side-scan sonar. By using sonar, the system receives echoes from underwater. The depth of the water is then calculated by measuring the speed and time of the echo energy signal propagation [17]. This process enables the acquisition of underwater topographic data. Next, the data are imported into the Southern CASS11.0 software to build a digital terrain model (DTM) and generate contour lines. A 1:500 underwater topographic map is created based on these contour lines. The underwater topographic map is shown in Figure 13a. By utilizing CASS along with the underwater topographic map, a rapid generation of the underwater topographic model is achieved. Finally, the Geomagic Wrap2021 software is used to refine the underwater topographic model by clipping boundaries, texture mapping, and other refinements and to export it in FBX format. The underwater terrain real 3D model is shown in Figure 13b.

### 4.4. The Fusion of Three-Dimensional Model of Full Space Reality

The constructed 3D models mentioned above have characteristics such as inconsistent format and large data volume. Traditional software often faces challenges in simultaneously loading and integrating these models, as well as slow loading speeds. To overcome these challenges, AGI has developed a web-based 3D digital globe rendering engine called Cesium. Cesium integrates and loads models of different formats through format conversion. It improves the loading speed of 3D models on the web by utilizing lightweight techniques and data service publishing [18]. Therefore, utilizing the Cesium platform to integrate and load the 3D models constructed in this article is a good choice.

**Figure 14:**
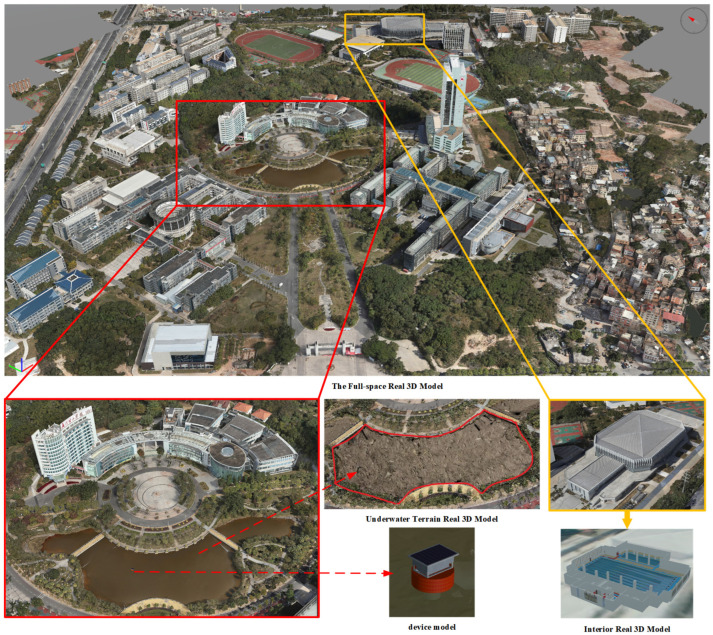
Results of full-space 3D model fusion.

In this article, the CesiumLab tool in the Cesium platform is first used to convert the aforementioned 3D model files in osgb, obj, and FBX formats to the 3DTiles format. Then, the model files are processed and published to make them lightweight. After the lightweight processing, the 3D models maintain their visual quality while reducing the file size, thus speeding up the loading of models in the browser. The osgb campus oblique model contains geolocation information, and the generated 3DTiles file, after data processing, has the correct geospatial coordinate file. It can be automatically loaded into the virtual globe at the correct geolocation. On the other hand, the underwater terrain model in FBX format, as well as the swimming pool model and water quality monitoring equipment model in obj format, do not have geospatial information files. Therefore, manual adjustments are required. Based on the oblique model as the reference, by combining the underwater terrain model, indoor swimming pool model, and water quality monitoring equipment model, information such as root nodes, center points, and transformation matrices, translation, rotation, and height adjustments are performed to load them in the correct geolocation, achieving the loading and fusion of indoor-outdoor and underwater models. The fused results of the full-space 3D models are shown in Figure 14.

## 5. Three-Dimensional Water Quality Monitoring System

**Figure 15:**
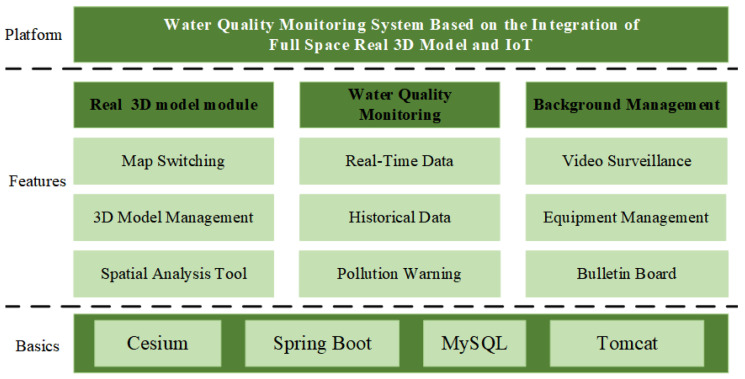
Functional structure of the system.

**Figure 16:**
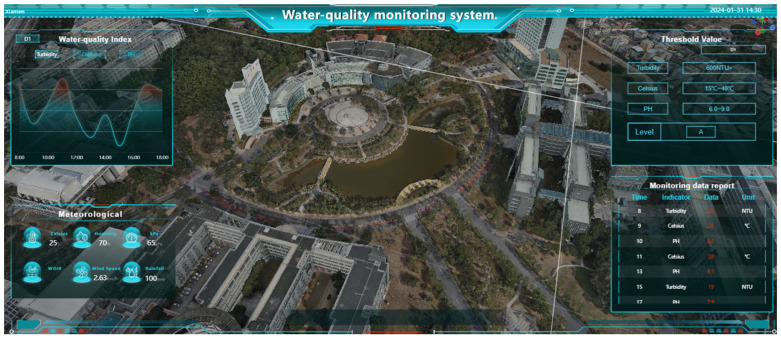
Main interface of the system.

In this paper, the SMM (Spring+SpringMVC+MyBatis) framework is used to complete the above obtained IOT data and full-space real 3D data fusion and visual interface design, and the system is connected to video monitoring data to increase the richness of system data. The system function modules include real 3D module, a water quality monitoring module, and a background management module. The detailed functions of each module are shown in Figure 15, and the main interface of the system is shown in Figure 16.

### 5.1. Real 3D Model Module

**Figure 17:**
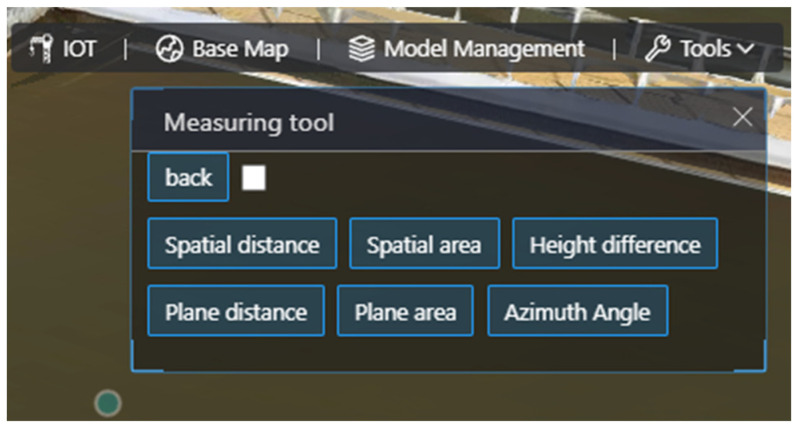
Three-dimensional module function diagram.

The real 3D module includes 2D/3D map switching, 3D model management, and spatial analysis tools. The 2D/3D map switching enables the display of multiple thematic maps and basic browsing operations such as switching, rotating, and roaming between 2D and 3D spaces. The 3D model management module displays each 3D scene of the system from large to small and from coarse to fine and shows the spatial situation and texture details of the study area in a real and 3D way, which brings users a real and 3D feeling. The spatial analysis tools allow for the management of model data in the research area, attribute queries, spatial measurements, and positioning operations. This module initially realized the digital mapping from physical space to virtual space, providing high-precision and high-quality local data for early-stage geographic and terrain surveys, as well as later stages of water pollution source tracing and ecological restoration planning. The 3D module function is illustrated in Figure 17.

### 5.2. Water Quality Monitoring Module

**Figure 18:**
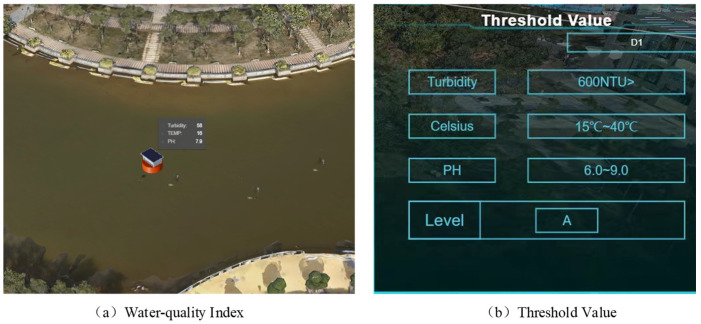
Function diagram of water quality monitoring module.

The water quality monitoring module is mainly responsible for the real-time and dynamic display of water quality information. Its functions include the display of water quality sensing information, management of historical data, and water quality alert notifications. The 3D visualization platform accesses the OneNet database through API, obtains water quality data and GNSS positioning data, and assigns the data to the water quality monitoring device model so that the monitoring device model and water quality monitoring data are dynamically presented in the 3D visualization platform. It also supports the retrieval of historical information within a certain time period, allowing for detailed observation of specific situations during that time period and providing users with an analytical decision-making basis. At the same time, administrators can customize the threshold range for water quality parameters. Once the monitoring devices detect water quality exceeding the safe range, the system will send alert messages to notify the administrators. The water quality monitoring module’s functions are illustrated in Figure 18.

### 5.3. Background Management Module

**Figure 19:**
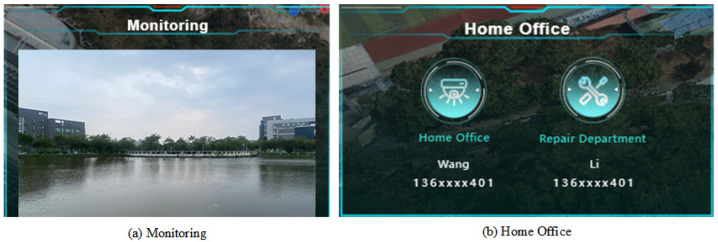
Function diagram of video surveillance and user management module.

The background management module includes video surveillance, monitoring device management, and task deployment. The video surveillance system connects the network monitoring data to the access system and displays it on the same screen as the real-time water quality monitoring data. It presents a diversified view of the monitoring area, assisting in the collaborative analysis of water quality data and supporting functions such as monitoring device management and task deployment. The video surveillance and user management module is illustrated in Figure 19.

## 6. Water Quality Data Analysis

This paper refers to the water quality evaluation method of the literature, “Multi-Sensor Data Fusion Technology Based on NB-IoT Environment Monitoring” [19], to evaluate the acquired water quality data. Firstly, the monitoring data from 15 June to 25 July in the study area were collated as an example, and the average value of water quality parameters was collated and calculated in ten days. The water quality data are shown in Table 2.

This paper determines the weights of different factors based on the entropy information scheduling method [18]. Firstly, the mathematical model is established by MATLABR2022b to obtain the relevant weight $A=\left\{\begin{array}{ccc} a_{1} & a_{2} & a_{3} \end{array}\right\}=\left\{\begin{array}{ccc} 0.1868 & 0.6269 & 0.1863 \end{array}\right\}$ , where *a*_1_ represents the weight value of temperature, *a*_2_ represents the weight value of turbidity, and *a*_3_ represents the weight value of PH.

A rating sheet is a collection of comment levels consisting of various general evaluation results that an evaluator can perform on an evaluation object. The result of classification is based on the “Surface Water Environmental Quality Standard” (GB3838-2002) [20] and the relevant statistics of the park responsible person, and the water quality is divided into four levels, A, B, C, and D, which are used to judge the degree of environmental quality. The evaluation grading is shown in Table 3.

Secondly, the factor set $U=\left\{\begin{array}{ccc} u_{1} & u_{2} & u_{3} \end{array}\right\}$ is listed, where *u*_1_ represents the temperature evaluation factor, *u*_2_ represents the turbidity evaluation factor, and *u*_3_ represents the PH evaluation factor. Next, the membership degree *r_i_* of each element *u_i_* in the factor set was calculated by the Gaussian membership function, and the membership degree set of each evaluation factor was used to form the single factor matrix *R_i_* of water quality evaluation. Its expression is as follows:

$$y=e^{-[\left(x-b\right)/\sigma ]2}\;\;\;\;\;\;-\infty <x<+\infty$$

where *x* is the evaluation factor *u_i_*, *b* is the standard value of the corresponding evaluation table of each water quality (as shown in Table 3), and σ is the standard deviation of the corresponding evaluation set of each water quality.

Finally, the fuzzy relation matrix is obtained as follows:

$$R_{1}=\left[\begin{array}{cccc} 0.9426 & 0.8754 & 0.2513 & 0.0822\\ 0.9993 & 0.9492 & 0.4771 & 0.0380\\ 0.9523 & 0.7665 & 0.2084 & 0.0723 \end{array}\right]\;R_{2}=\left[\begin{array}{cccc} 0.2170 & 0.6740 & 0.9672 & 0.7080\\ 0.9947 & 0.9680 & 0.5170 & 0.0451\\ 0.8225 & 0.9168 & 0.3452 & 0.1410 \end{array}\right]\;R_{3}=\left[\begin{array}{cccc} 0.3156 & 0.8048 & 0.8881 & 0.5700\\ 0.9933 & 0.9711 & 0.5248 & 0.0466\\ 0.2084 & 0.7665 & 0.9523 & 0.7066 \end{array}\right]$$

Based on the weighted parameters, a quantitative analysis of water quality was conducted, revealing the water quality parameters for the period from 15 June to 25 June as follows:

$Y_{1}=A\cdot R_{1}=\left[\begin{array}{cccc} 0.9800 & 0.9014 & 0.3849 & 0.0526 \end{array}\right]$ , *Y*_1_(*max*) = 0.9800. It can be seen that the water quality evaluation grade for this period is A.

The water quality parameters for the period from 1 July to 10 July are

$Y_{2}=A\cdot R_{2}=\left[\begin{array}{cccc} 0.8173 & 0.9035 & 0.5691 & 0.1868 \end{array}\right]$ , *Y*_2_(*max*) = 0.9035. It can be seen that the water quality evaluation grade for this period is B.

The water quality parameters for the period from 16 July to 25 July are

$Y_{3}=A\cdot R_{3}=\left[\begin{array}{cccc} 0.7205 & 0.9019 & 0.67723 & 0.2673 \end{array}\right]$ , *Y*_3_(*max*) = 0.9019. It can be seen that the water quality evaluation grade for this period is B.

According to the data provided by the park leader, the water quality level was C before the 3D visualization water quality system was deployed. Therefore, the system can effectively assist in the improvement of small and micro-water quality.

## 7. Conclusions

Based on the current demand for ecological restoration of small and micro-ecosystems and water quality monitoring and management, this paper proposes a solution that combines IoT with full-space real-world 3D technology. It utilizes GNSS, multi-source sensors, and NB-IoT wireless communication technology to achieve real-time acquisition and wireless transmission of water environmental information, and it uses the OneNet cloud platform server to efficiently manage massive IoT data. Through technologies such as unmanned aerial vehicle (UAV) oblique photography, unmanned boat underwater measurement system, and 3D laser scanning, complete spatial data imagery can be quickly obtained. Then, DP Modeler, SketchUp, and other software are used to restore the scene, refine individual elements, and create detailed indoor models. The full-space data volume is made lightweight, and model fusion operations are performed on the Cesium platform. Finally, based on IoT sensing data and full-space real-world 3D data volume, a real-world 3D campus water quality monitoring platform is designed using the SMM framework.

The system presents campus buildings, environments, and monitoring equipment in 3D form, reproducing real campus scenes in a simulated space and integrating dynamic water quality sensing data from the IoT, achieving real-time correlation and communication between the digital space and the real space. And through the comparison of water quality data in the early and late stages, it can be seen that under the monitoring of “Water Quality Monitoring system Based on the Integration of Full-Space Real 3D and IoT” small and micro-water quality has been effectively improved. Furthermore, the system can assist managers in non-contact ecological environment surveys and efficient data integration in the early stage. In the middle stage, it can monitor water quality and water environmental information in real time from a three-dimensional perspective in the full space. In the later stage, it can combine historical data and spatial geographic information to investigate the origin of pollution problems and develop plans for remediation and management. The proposed solution in this paper provides a new method and new ideas for monitoring and treating small and micro-water bodies.

## Figures and Tables

**Table 1:** Water quality sensor main parameters table.

Sensor	Range of Measurement	Range of Error	Voltage
PH Sensor	0~14 pH	±0.15 pH	10~30 V
Turbidity Sensor	0~4000 NTU	±5%FS (25 °C)	10~30 V
Temperature sensor	−55 °C~+125 °C	±0.5 °C	3.3 V~5.5 V

**Table 2:** Water quality data table.

Date	Temperature/°C	Turbidity/NTU	PH
6.15–6.25	27.2	27.8	8.2
7.01–7.10	32.1	43	7.9
7.16–7.25	31.3	45.9	6.8

**Table 3:** Water quality indicator form.

Level	Temperature/°C	Turbidity/NTU	PH
A	26	20	8.5
B	29	100	7.5
C	33	300	6.5
D	35	600	6.0

## Data Availability

Data are contained within the article.
